# Anti-Smith Antibody Positivity Without Lupus in the Setting of Lung Cancer

**DOI:** 10.7759/cureus.8298

**Published:** 2020-05-26

**Authors:** Homa Timlin, Kirthi Machireddy, Jennifer Shiroky, Srekar Ravi, Uzma Haque

**Affiliations:** 1 Rheumatology, The Johns Hopkins University School of Medicine, Baltimore, USA; 2 Osteopathic Medicine, Edward Via College of Osteopathic Medicine, Blacksburg, USA; 3 Internal Medicine, Bayview Medical Center, Baltimore, USA; 4 Medicine, Charles E. Schmidt College of Medicine, Florida Atlantic University, Boca Raton, USA

**Keywords:** anti-smith antibody, cancer, systemic lupus erythematosus

## Abstract

The interpretation of positive serologic findings in cancer sera remains controversial. Selected antinuclear antibodies (ANAs), such as anti-double-stranded deoxyribonucleic acid (dsDNA) and anti-Smith, are highly specific for the diagnosis of systemic lupus erythematosus (SLE). On the other hand, the ANA titer is sensitive but not specific for SLE and has been reported in healthy individuals, various infections, other autoimmune diseases and cancer. We report for the first time positive anti-Smith antibody in two patients without lupus in the setting of lung cancer.

## Introduction

Systemic lupus erythematosus (SLE) is a chronic autoimmune disease characterized by the production of autoantibodies and the deposition of immune complexes, affecting a wide range of organs. The diagnosis of SLE is based on specific clinical and laboratory criteria after excluding alternative diagnoses [1]. In this report, we describe two individuals with lung cancer who were positive for anti-Smith antibody without meeting the criteria for SLE, during evaluation for unexplained weight loss. Both patients denied inflammatory arthralgia, mucosal ulcers, alopecia, photosensitive skin rash, Raynaud’s phenomena or sicca symptoms.

## Case presentation

Patient A is a 59-year-old female with a history of diabetes, hypertension, hyperlipidemia, tobacco use and stage 3B, poorly differentiated carcinoma with glandular and neuroendocrine features treated with two courses of carboplatin/etoposide therapy. Her labs revealed positive antinuclear antibody (ANA) at a titer of 1:320 in a speckled pattern, positive Smith at a titer >694, positive RNP, positive anti-Ro52 IgG and low C3. C4 was normal and anti-dsDNA, anti-histone, anti-La and anti-cardiolipin antibodies were negative. Follow-up anti-Smith antibody after chemotherapy demonstrated a reduction in the anti-Smith titer, IgG 349.9.

Patient B is a 63-year-old male with a history of tobacco use and Crohn’s disease who was hospitalized with hemoptysis and subsequently diagnosed with lung adenocarcinoma. His labs were checked before this admission, and these showed positive ANA, anti-Ro60 IgG, anti-RNP and anti-Smith at a titer of 38. Serum C3 and C4 were normal, and anti-La and anti-dsDNA were negative.

## Discussion

Autoantibodies targeting intracellular antigens are the hallmark of SLE. Selected ANAs, including anti-double-stranded deoxyribonucleic acid (dsDNA) and anti-Smith, are highly specific for the diagnosis of SLE. However, anti-Sm antibodies lack sensitivity and are seen in only approximately 30% of patients with SLE. Concerns have been raised about the utility of testing antibodies due to decreased pretest probability in patients with various non-rheumatologic conditions.

The significance of autoantibodies in the serum of patients with cancer, or the possibility of cancer-related etiology of SLE and other autoimmune diseases, has yet to be fully elucidated. Studies have focused primarily on identifying antibodies as biomarkers rather than investigating the underlying causes of their production. Approximately 30% of all cancer patients have circulating ANAs in their sera [2]. The presence of autoantibodies may represent an effective immune response to the tumor, as suggested by a higher survival rate in cancer patients who are ANA-positive [3]. Their production can precede clinical confirmation of a tumor by several months or years [4]. Furthermore, studies have demonstrated positive anti-Smith antibodies in cancer patients, such as gastrointestinal, ovarian, renal cell carcinoma and hematological malignancies [5]. 

Understanding the causes of antibodies production in different cancers may lead to the development of novel therapeutic agents. In patients with lung cancer and without lupus, the significance of positive anti-Smith antibody and decrease in the titer of antibody after therapy remains unknown. These findings may have implications in helping to understand the mechanisms that dysregulate autoantigen production and drive autoantibodies in cancer patients without an autoimmune connective tissue disease.

## Conclusions

Anti-dsDNA and anti-Sm antibodies are clinically thought to be highly specific for SLE. We report for the first time that isolated seropositivity for anti-Smith antibody can occur in the setting of lung cancer, and our cases add to others that question the specificity of anti-Smith antibodies in SLE.
